# A kinematic dataset of locomotion with gait and sit-to-stand movements of young adults

**DOI:** 10.1038/s41597-024-04020-6

**Published:** 2024-11-09

**Authors:** Simon Hanisch, Loreen Pogrzeba, Evelyn Muschter, Shu-Chen Li, Thorsten Strufe

**Affiliations:** 1 Technische Universität Dresden, Centre for Tactile Internet with Human-in-the-Loop, Dresden, 01062 Germany; 2 Karlsruhe Institute of Technology, Computer Science, Karlsruhe, 76131 Germany; 3 Technische Universität Dresden, Chair of Lifespan Developmental Neuroscience, Dresden, 01062 Germany; 4 Technische Universität Dresden, Research Hub 6G-life, Dresden, 01062 Germany

**Keywords:** Anatomy, Computer science, Machine learning, Scientific data

## Abstract

Kinematic data is a valuable source of movement information that provides insights into the health status, mental state, and motor skills of individuals. Additionally, kinematic data can serve as biometric data, enabling the identification of personal characteristics such as height, weight, and sex. In CeTI-*Locomotion*, four types of walking tasks and the *5 times sit-to-stand test* (5RSTST) were recorded from 50 young adults wearing *motion capture* (mocap) suits equipped with *Inertia-Measurement-Units* (IMU). Our dataset is unique in that it allows the study of both intra- and inter-participant variability with high quality kinematic motion data for different motion tasks. Along with the raw kinematic data, we provide the source code for phase segmentation and the processed data, which has been segmented into a total of 4672 individual motion repetitions. To validate the data, we conducted visual inspection as well as machine-learning based identity and action recognition tests, achieving 97% and 84% accuracy, respectively. The data can serve as a normative reference of gait and sit-to-stand movements in healthy young adults and as training data for biometric recognition.

## Background & Summary

Kinematic data of human motion is a rich source of information that has long been captured for use in the medical field^1^, and now, with increasing digitization, is being captured more and more for use in Mixed Reality (MR) and the Tactile Internet^2^. For applications in both areas, movements need to be captured in great detail, including their kinematics, to create a digital twin of that person, which could then be used to interact with the digital world. Examples of uses of kinematic data in this context include motion control of robots, animation of digital avatars in virtual meetings, or gesture control for applications. Consequently, increasingly more kinematic data is being collected outside the laboratory, providing insights into how individuals walk in everyday situations and enabling the development of interventions tailored to real or digitized scenarios. In addition, kinematic gait data provide valuable information about an individual’s locomotion, but also more generally about their functional abilities and limitations. Such data can assist the assessment of balance, sensorimotor coordination, and overall mobility, which are essential for activities of daily living and can serve as indicators of basic health status. A recent study used kinematic data to derive gait speed and spatiotemporal gait parameters in a young population, which made a valuable contribution for establishing reference values in gait assessments^3^. Furthermore, this type of data promotes the exploration and testing of gait features through machine learning, thereby facilitating the development of clinical applications^4^. Indeed, kinematic analyses of gait and other movement data gain increasing importance in clinical applications. By analysing gait pattern and stand-to-sit exercises^5–7^, clinicians can identify abnormalities^8–13^, track process^14,15^ or assess functional outcomes^16,17^, and make informed decisions regarding interventions and rehabilitation strategies (e.g. for designing auditory feedback in gait training for patients post-stroke^18^ or after unilateral hip arthroplasty^19^).

In recent years it has been shown that gait, and motion data in general, can be used as a biometric factor to identify individuals and infer private information about them^20^. Recognizing people from their gait patterns in 2D videos is a field of research in its own right^21–23^. A recent extension of this research is to investigate gait recognition on 3D mocap data^24,25^. In addition, motion data can be used to infer attributes such as a person’s sex^26^ and age^27^. Due to these inference of identity and personal attributes motion data can further be used to develop and evaluate new privacy protecting techniques^28^.

In order to contribute to both the medical and biometric aspects of using motion data, we recorded a new dataset in a young adult sample. Our dataset has the advantages of capturing motion of the entire body with good precision in a relatively large sample of young adults performing four different types of gait movements and multiple repetitions of the sit-to-stand task. Such data allow intra-individual variability and its variations across individuals be taken into account for training machine learning methods. These features are better suited for medically relevant applications for which full body motion capture with a good accuracy are necessary for assessing functional status of different body parts. Further, having a large of number of participants in performing different types of tasks with high numbers of repetitions is important to train machine learning algorithms in identifying biometric features of individuals.

For motion tracking, we utilized an IMU mocap suit as it is a good compromise between setup complexity and tracking quality for full body motion. An IMU sensor consists of an accelerometer, gyroscope, and magnetometer that estimate the position and orientation of the IMU sensor. IMU based motion tracking offers a good accuracy of relative movements of the individual body parts. However, compared to optical marker-based tracking as the gold standard of motion capture, IMU motion capture is less accurate for positions and orientations. The difference between the two approaches is nevertheless slight (e.g., around +/−2 degrees, see Mihcin^29^ and later in discussions). An important benefit of IMU based tracking over optical tracking is that it is not limited by a certain spatial volume in which the recording takes place, which makes it easier to capture longer sequences (e.g. Horst *et al*.^24^ capture only a single gait cycle per recording) or to avoid the need of using treadmills (e.g. Troje *et al*.^30^). In addition, the setup time of a recording session is much shorter, as putting on an IMU suit is much faster than sticking a full set of markers to the bodily landmarks of a participant and the calibration of the IMU system requires also less time.

In summary, kinematic data has been instrumental in advancing research in various fields, including rehabilitation, biomechanics, sport science and privacy science. With this new dataset we aim to facilitate the exploration of new insights into human locomotion, and the development of advanced analysis techniques. Thus, the present dataset can be used both for reference in medical analysis for young healthy adults and in the field of biometric recognition for both identity and personal attributes. In addition, the dataset can be used to develop new privacy protections to prevent inference of identity and personal attributes^28^.

## Methods

### Participants

**Fig. 1:**
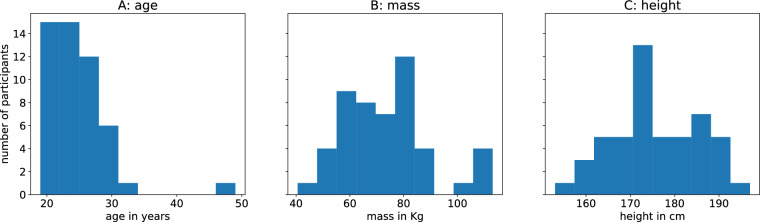
Overview of the demographics of the participants as histograms. Panel A shows the age distribution in years, Panel B shows the mass distribution in kg, and Panel C shows the height distribution in cm.

Data was recorded from 50 young adults (28 male, 22 female; age mean 24.3 years, std. 4.7 years; mass mean 73.5 kg, std. 16.2 kg; height mean 175.5 cm, std. 9.8 cm). An overview of the demographics can be seen in Fig. 1. All participants gave written informed consent and agreed to the publication of the study data. The study was approved by the Ethics Committee of the Technical University of Dresden (SR-EK-5012021) and was conducted in accordance with the tenets of the Declaration of Helsinki. Data were pseudonymized and a unique ID was assigned to each participant.

### Acquisition Setup

The dataset was recorded for each participant individually in the laboratory of Karlsruhe Institute of Technology. The single recording session on average lasted for about 60 minutes. Before the recording started, we marked the walking area of 3.5 m with two red crosses on the floor.

#### Anthropometric Data

Anthropometric data was recorded for all participants using an anthropometric grid^31^ for total height and arm span of the participants. Additionally, key anthropometric body measurements (i.e., shoulder height, shoulder width, pelvis height, pelvis width, knee height, foot length, and manus length) were taken manually with a generic measuring tape and ruler (stated accuracy  ± 0.9 mm) following the anthropometric measurement template of the manufacturer of the mocap suits. This data was then used to create a body profile for each participant. Finally, a standard personal scale (Huawei AH100) was used to determine the weight of each participant (wearing a mocap suit and without shoes). The weight values are given with the mocap suit (about 1.2 kg) included.

#### Kinematic Data

**Fig. 2:**
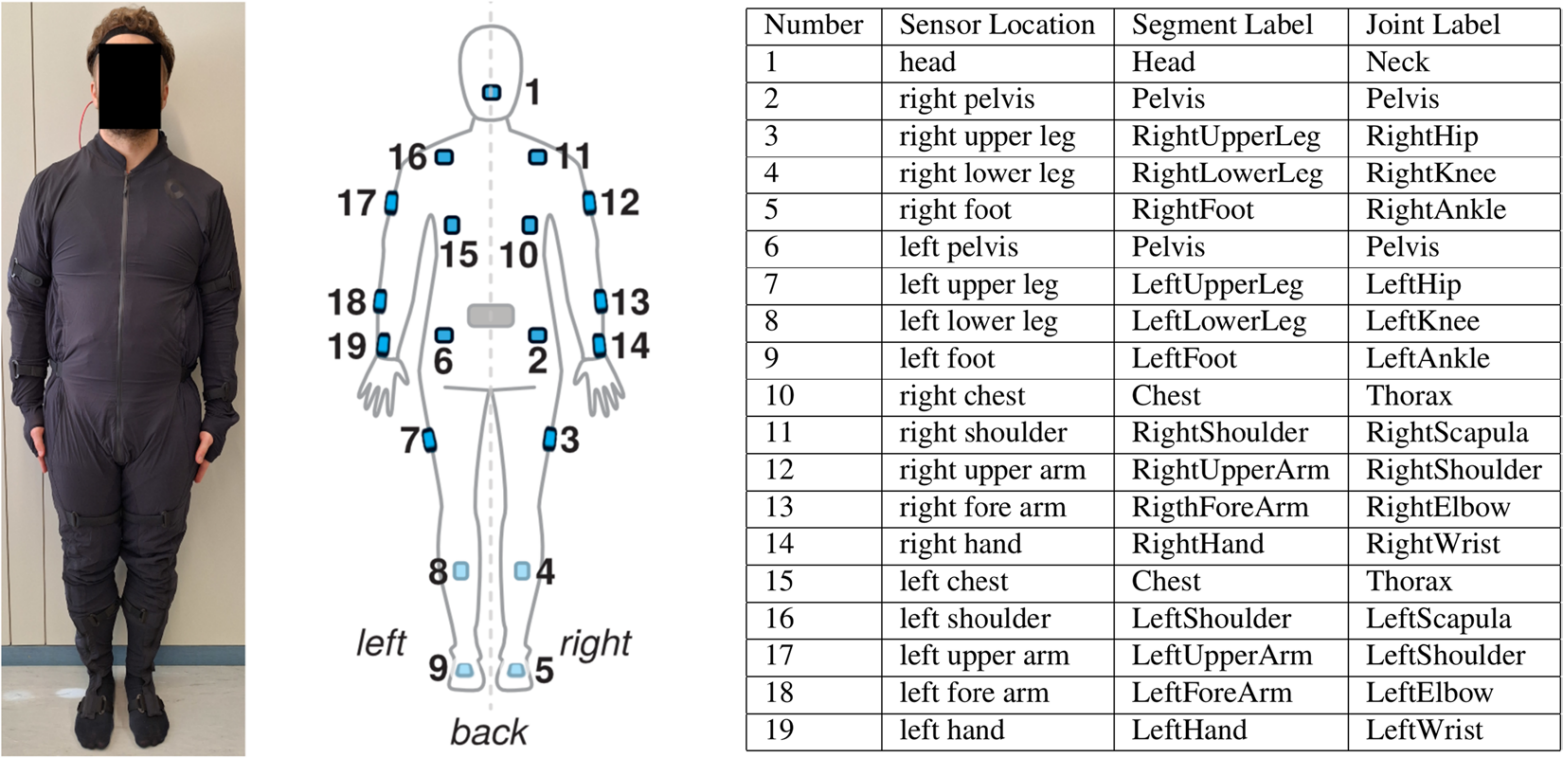
Participant wearing Rokoko Smartsuit Pro 1 in calibration pose (left) and schematic sensor locations (middle andright) on the suit depicted from a posterior view. The sensors are color-coded, with light blue sensors (4, 5, 8, 9) positioned anteriorly. The segment and joint labels, provided by Rokoko Electronics Inc., are named according to the tracked centers of body segments, with rotation axes conforming to the standardized Joint Coordinate System (JCS) defined by the International Society of Biomechanics (ISB)^32,33^. A full overview of the channels of each recording can be found in the *_channels.tsv files.

Motion data were collected using the Smartsuit Pro 1 (see Fig. 2 left and middle panel) and Rokoko Studio (version 1.20.5r) mocap technology provided by Rokoko (Rokoko, Denmark, https://www.rokoko.com). The mocap suit contains a total of 19 IMU sensors (see Fig. 2 right), with one sensor each on the foot, shin, thigh, hand, forearm, upper arm, shoulder, and head. The remaining sensors are located on the torso, with two sensors each on the lower back and hips. The sensors are held in place by Velcro and the fabric of the suit. Mocap data is recorded at 100 Hz and transmitted via WiFi to the recording computer and then applied to the participants’ skeletal rig. The skeletal rig is a representation of the key body parts with the proportions of the participants’ body profile (described above).

### Acquisition Procedure

**Fig. 3:**
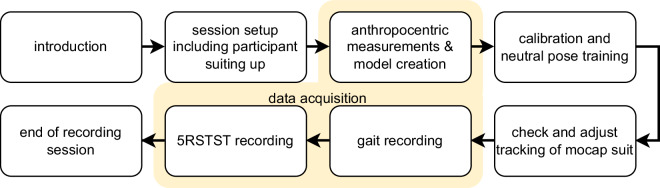
The study protocol.

Figure 3 offers a schematic overview of the study protocol. Before the data acquisition started, participants were asked to change in a thin layer of sports garments, over which they wore the mocap suit. A private changing room was provided. Participants were asked to perform the exercises without shoes, wearing socks only. The mocap suit’s sensor locations on the limbs were carefully adjusted to the participants’ individual body morphologies according to the manufacturer’s guidelines. Next, participants were familiarized with the mocap suit and technology and given a general overview of the types of movements they had to perform. They were informed about how the movements and anthropometry of their body would be recorded. We then proceeded with the collection of anthropometric measurements.

The data acquisition started with the actor profile set up in Rokoko Studio where participant-specific ID, demographics, and anthropometric measurements were recorded and the system was calibrated. The calibration pose (straight pose with legs, arms, hands and fingers straightened, feet hip-wide apart) was used to set up the initial sensor position to ensure correct motion tracking. The neutral pose (stand upright with upper body straight, feet foot-width apart, weight evenly distributed on both feet, arms hanging loosely) served as a neutral reference pose from which the movements for all walking exercises were initiated. While the calibration pose places great emphasis on the correct alignment and rotation of body segments, the neutral pose should reflect a natural and comfortable resting pose. Participants then performed a series of control movements covering the range of motion of multiple body parts (e.g., shoulder abduction and flexion, wrist flexion and extension, ankle plantar flexion and dorsiflexion) to ensure best mocap quality. If necessary, sensors were re-positioned and the calibration was restarted. For two participants (sub-K15 and sub-K35) minor recording errors persisted for their arms, as specified in the metadata of the participants.

**Fig. 4:**
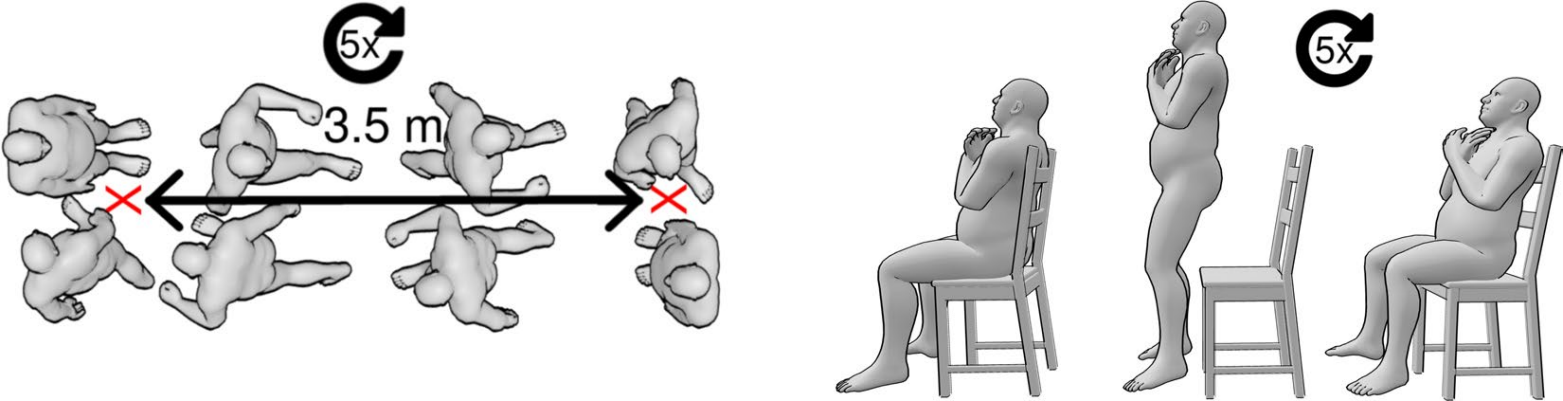
Schematic representation of a gait trial (left) shown from the top and 1RSTS trial (right) shown from the side.

Next, the participants were guided through the recording session, starting with the sequence of gait trials and finishing with the 5RSTST trials, in a predefined order (see also Table 1). Prior to each task, a mandatory calibration procedure was conducted to ensure accurate motion tracking. In order to capture potential intra-individual and inter-individual differences in gait movements, our comprehensive dataset includes kinematic data collected under four distinct conditions of walking. The first condition involved recording the natural walking speed and gait style of each participant at their preferred pace. In the second condition, participants were instructed to imagine being in a hurry, simulating a fast walking scenario. The third and fourth conditions focused on walking with an additional load at the participant’s preferred speed. Participants either carried a 5 kg backpack (total weight) or transported a standard bottle crate (measuring 400 mm x 300 mm x 270 mm, with a total weight of 5 kg evenly distributed). For all gait movements, participants were provided with explicit instructions to initiate from a neutral pose and walk between two points marked on the ground with red tape, positioned 3.5 m apart (see Fig. 4 left). Additionally, participants were instructed to maintain a forward gaze aligned with their walking direction. Participants were given specific instructions to execute a controlled turn at the marked points before commencing the return walk. Each gait task consisted of five back-and-forth walks between the designated points. The experimenter verbally counted the number of trials completed during each task. In the final round, participants were instructed to execute an additional turn, ensuring they returned to the original starting position and faced the initial direction.

**Table 1:** Overview of the resulting number of segments (*N*) per task after processing.

task	*N*_total_	*N*_average_ per participant	${{\bf{N}}}_{{\bf{\min }}}$ per participant	${{\bf{N}}}_{{\bf{\max }}}$ per participant
gait normal	1178	23.56	12	35
gait fast	846	16.92	9	23
gait bottle crate	1120	22.4	15	33
gait backpack	1029	20.58	11	30
1RSTS	499	9.98	9	11

Following the completion of the walking sequence, data collection proceeded with the 5RSTST trials (see also Fig. 3). Participants were provided with specific instructions to sit on a chair, crossing their arms in front of their chest, and perform rapid repetitions of standing up and sitting down (see Fig. 4 right) five times consecutively, without any intervals of rest. The execution time of each 5RSTST exercise was measured using a stopwatch, while the kinematic data was simultaneously captured using a mocap suit. Each participant performed this exercise twice.

### Data Processing

As part of the Rokoko Studio data recording pipeline, the position and orientation data was processed using the following filters. This involved the locomotion filter for all recordings that automatically aligns the feet sensor to the ground plane for movement phases with (assumed) ground contact. Additionally, a drift filter was applied for all gait trials to correct for position drift in the gait trajectories. Since IMU sensors only record the relative motion of each body part, the global position of the suit is estimated by summing up all relative movements beginning from the initial position (established during calibration). Over recording time, this method can lead to increasing positional inaccuracy, known as global drift. Since the start and end point of the walking course in the gait tasks was at the same position, this information was used to correct for global drift. Data was then exported in CSV format. The data includes the absolute positions (x, y, z) of the center of mass of all 17 body segments in meters and the relative orientation of each joint as recommended by the International Society of Biomechanics (ISB)^32,33^. The ISB standard defines the local axis system of joints in degrees with two fixed axes and one “floating" axis. In total, the data contains 17 6-dimensional (6D) points per time frame.

**Fig. 5:**
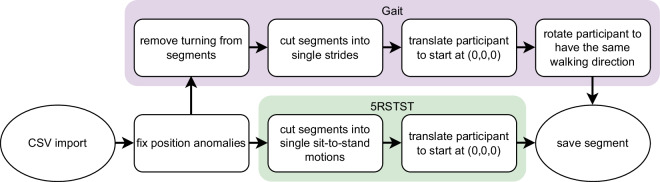
Data processing pipeline in custom Python script.

Finally, the data was processed using a custom Python (Python Software Foundation, USA, https://www.python.org) script^34^ (see Fig. 5). The data was visually inspected for noisy data segments and sensors. Anomalous sensor behavior was identified at individual time points within the position data of two participants (sub-K8, sub-K58), characterized by abrupt signal increases in multiple orders of magnitude. To remedy these artifacts, an interpolation approach utilizing the neighboring data points was implemented. Subsequently, we proceeded with cutting the recordings into individual segments. For the gait task a segment is a single gait cycle (as defined by Perry *et al*.^35^), and for the 5RSTST task it is a single 1RSTS repetition. A 1RSTS repetition begins with a participant sitting upright in a chair and ends with the participant reaching the same sitting position after standing up. This segmentation step allowed for a more granular analysis and examination of specific movements within each segment.

Data segmentation was based on the characteristics of the positional data signal. For tailoring the segmentation approaches to the characteristics of the different tasks, selected positional values of the signal were processed and searched for distinct events, e.g., peak values, to subsequently crop the signal into segments. The positional values are mapped to a coordinate system with the starting point of the movement is set to the coordinate origin (set during calibration), with the x-axis reflecting the position in the transverse plane (e.g., shoulder positions left and right to the median plane), the y-axis describing the heights of body parts (e.g. the head height), and the z-axis displaying the translation in the median plane (e.g., a forward step). While straight walking, the distance between the shoulder x positions (reflecting the approximate width of the shoulder girdle) stays constant. The distance changes at the turning points of the walking course as the participant rotates around its own body axis, from positive to negative or vice versa.

The resulting segments were then cut into single gait cycles, i.e., the feet end in the same position as they started. We chose gait cycles as they contain all gait phases performed while walking. The gait cycles were identified by detecting the largest distance between the absolute z-positions of the participants’ feet. As a final processing step, we normalized all gait cycle segments to start at the same coordinate point by setting the positional data of the hip at the starting frame of the segment as the new coordinate origin of the coordinate system (with translating all positional values accordingly). Next, we applied a rotation step to the positional values of each segment so that all segments reflected the same walking direction. This processing step was necessary, since the participants were walking back and forth at the walking course, resulting in different walking directions and corresponding positional values.

For the 5RSTST, we identified a single segment by observing the participant’s head position. We detected peak values in the (y, z) values of the head position, since these can be used to distinguish the sitting from the standing position. We automatically sliced them into five repetitions (integrating the standing and sitting phases into one repetition). As with the gait data, we used the first hip position of each segment as the coordinate origin for this segment.

An overview of the resulting number of segments processed can be found in the Table 1. Note that the resulting amount of segments for each participant and task varies due to several factors: faster walking results in fewer segments per participant, individual physiology affects the number of segments per person, and because we excluded incomplete gait cycles. For 5RSTST, we also found an incorrect execution of the task, as one participant performed more repetitions than instructed.

## Data Records

We provide the CeTI-*Locomotion* dataset^34^ on the figshare data exchange platform (https://figshare.com/). The dataset is formatted according to the BIDS^36,37^ standard (version 1.9) and is provided in both raw format (as exported from Rokoko Studio) and processed format (as described above). The repository contains a readme, license, BIDS dataset descriptor, and the Python code used for processing and technical validation. The *participants.tsv* file stores the metadata for all participants, including their age, mass, height, anthropometric data, and 5RSTST completion time. Each subject folder contains the raw motion data for all tasks. The motion data is stored as a tab-separated values file (*_motion.tsv) accompanied by a metadata file (*_motion.json) and a channel descriptor of the motion file (*_channels.tsv). The processed data is located in the derivatives folder and follows a similar structure, but split into the processed segments.

## Technical Validation

**Fig. 6:**
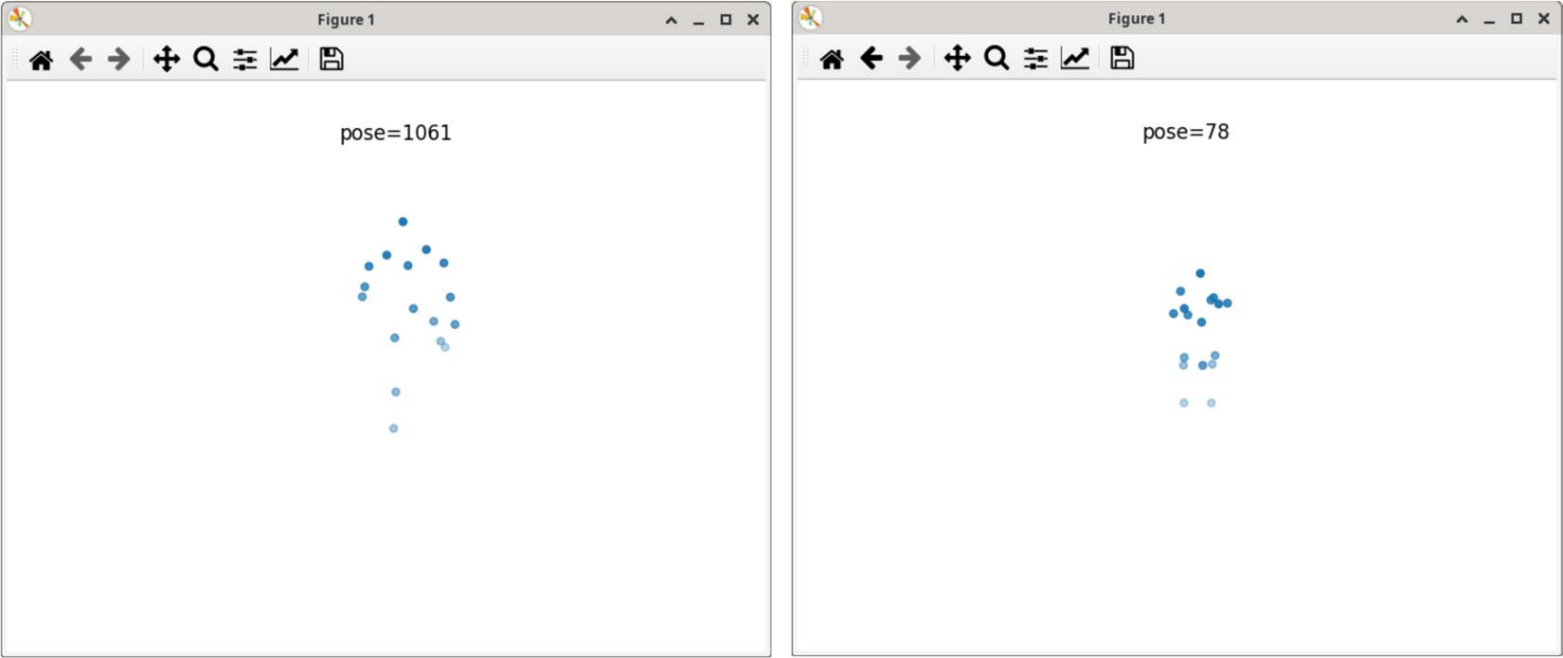
Example of a visual inspection rendering of a gait (left) and 5RSTS (right) recording.

**Fig. 7:**
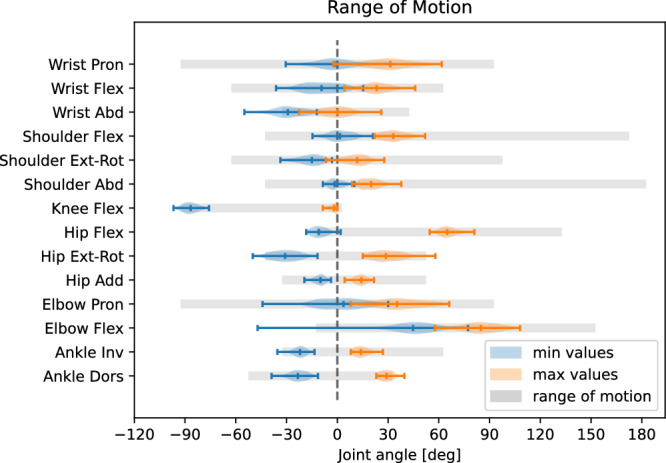
Distribution of average minimum and maximum joint angles for all participants, as calculated across all raw recordings per participant. Anatomically typical range of motion (ROM) values reported in prior research^38^ are shaded in gray.

In general, the suitability of using the Rokoko Smartsuit for motion data recording has been validated by previous work. Mihcin *et al*.^29^ reported good agreement between the Smartsuit and an optical tracking system, with a maximal bias range -1.48 – 2.22 degrees for knee flexion and for hip abduction-adduction. We performed two types of validation, one prior to recording our data. Here, we visually inspected the tracking performance of the Rokoko Smartsuit by having participants perform static control poses, such as a T-pose (with 90 degrees shoulder abduction) or lifting their legs. Only when we found the tracking to be accurate, we did start recording the mocap data. The second validation was done after exporting and processing the data into Python. In a first step, we rendered the position data of a random selection of segments to visually inspect a natural-looking motion execution within the anatomically possible range of motion (ROM) (see Fig. 6). Following this initial manual inspection, we evaluated whether the minimal and maximal joint angles fell within the anatomically feasible ROM. For each participant, we first calculated the minimal/maximal joint angles across all motion segments, for each of the six raw recordings separately (5RSTS is split into two recordings). We then averaged the minimum and maximum joint angles for each participant across recordings. Figure 7 depicts the distribution of mean minimal (in blue) and mean maximal (in orange) joint angles for all participants in comparison to reference range of motion values from Ryf and Weymann^38^ (in gray). Note, that we only plot joint angles for which reference values are available. For most joints the values fall within the expected ROM. Outliers in knee flexion, hip external/internal rotation, and ankle inversion/eversion could be attributed to inaccurately executed calibration poses or faulty sensor measurements (e.g., for elbow flexion in sub-K9). Additionally, joint angles for wrist abduction exceeded the normal ROM for some participants, likely due to inconsistent positioning of the wrist during calibration.

In addition, a classification analysis was performed to assess the suitability of the processed data for machine learning applications. Our model setup is comparable to Horst *et al*.^24^ and we have provided the our source code as part of this Data Descriptor (see Section Code Availability). For classification, we employed a support vector machine (SVM)^39,40^. The SVM was utilized to classify the movement tasks, the sex of the participants, and the participants’ identities. We chose a SVM with a radial basis function (RBF) kernel, a regularization parameter (*C*) of 1, gamma which is 1/ (n_features*Var(*X*)). As a preprocessing step for machine learning, we standardized the length of each motion segment by resampling them to contain exactly 100 frames. Additionally, we concatenated the time frames, which consisted of 17 6D points, into a single vector representation, resulting in a vector of size 10200. To further prepare the data for machine learning algorithms, we applied scaling techniques to normalize the values within each dimension of the data. By scaling the values to a range between 0 and 1, we ensured that all features contributed equally to the learning process, regardless of their original magnitude. To reduce the dimensionality of the dataset and capture the most relevant information, we employed principal component analysis (PCA)^41^. This technique transformed the high-dimensional feature space into a lower-dimensional representation while preserving the most significant variation in the data. This resulted in a segment vector of 3342 to 3363 elements (depending on how the split was performed), with the first 10 PCA components explaining 69%−68% of the variance. The kinematic data was split into a test (20%) and a training (80%) dataset. For the identification classification we split segment-wise, i.e. 80% of a participant’s segments are in the training dataset and the remaining 20% are in the test dataset. For sex and modality classification, we split participant-wise, with all segments of a participant in either the training or test dataset, with 80% of the participants in the training and 20% in the test dataset. On the training dataset we performed a stratified 10-fold cross-validation using balanced accuracy (mean of the recall per class) as the metric to select the most appropriate model before testing it on the test dataset.

**Fig. 8:**
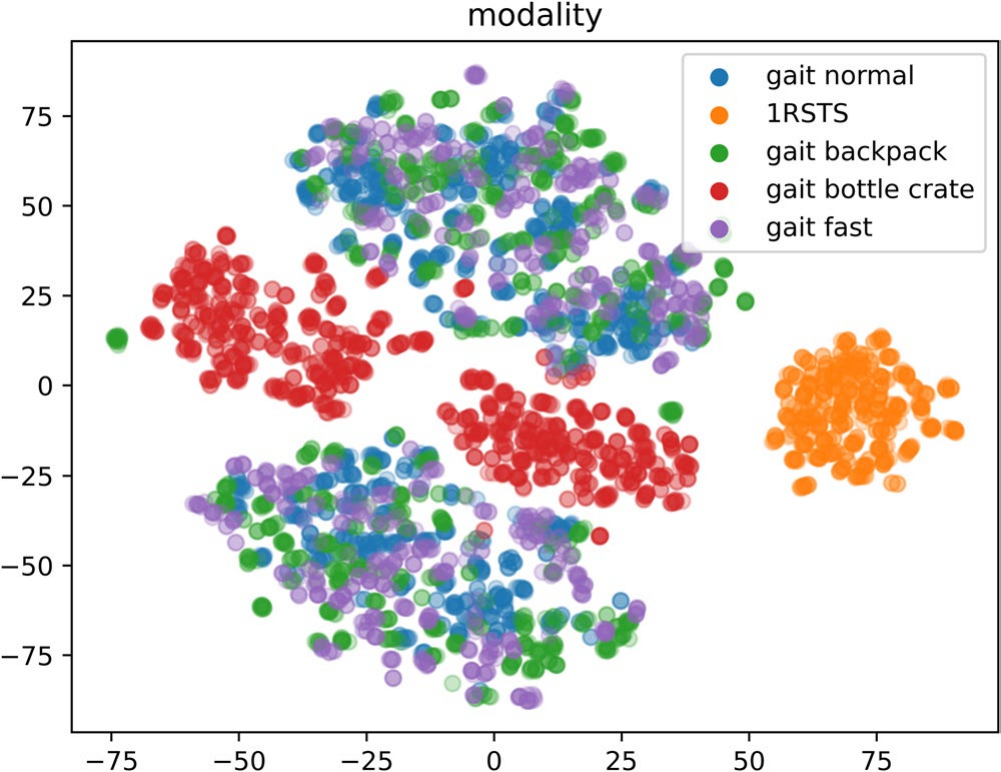
t-SNE plot colored for different motion tasks.

The metrics accuracy (number of correct predictions divided by total predictions) and F1-scores^42^ were used to evaluate the performance of classification models and are reported in Table 2. As both metrics achieve comparable results we only discuss the accuracy in the following. Our classification model achieves 97% identification accuracy, a 81% sex recognition accuracy, and a 84% action recognition (gait normal: 90%, gait fast: 93%, gait backpack: 88%, gait bottle create: 97%, and 1RSTS: 100%). For action recognition, a similar result can be found when we plot the actions in two dimensions using t-SNE^43^ (perplexity 30), see Fig. 8. t-SNE is a stochastic method for visualizing high dimensional data as two dimensional data points while preserving the similarity of the data points. The clusters of the 1RSTS and gait bottle crate segments are clearly distinguishable from the remaining task segments. The clusters for the remaining task segments overlap and cannot be clearly separated, showing their similarity. Note that for each gait modality we see two clusters. The plot is in line with the results of the classification experiments, as 1RSTS and bottle crate gait are easily separated from the other modalities due to their uniqueness, while the separation of the remaining gait modalities is more difficult.

**Table 2:** The classification results given as accuracy and F1-score for different attributes, we also report the number of classes (*C*) and number of segments per class (*N*) used for the classification.

classification	accuracy	f1 score	*C*	*N*_*average*_	*N*_*min*_	*N*_*max*_
identity	97%	97%	50	93.44	61	129
sex	81%	81%	2	2336	2046	2626
action all	84%	85%	5	934.4	499	1178
1RSTS	100%	100%	2	2336	499	4173
gait normal	90%	83%	2	2336	1178	3494
gait fast	93%	78%	2	2336	846	3826
gait backpack	88%	72%	2	2336	1029	3643
gait bottle crate	97%	94%	2	2336	1120	3552

## Related Datasets

Comparing our dataset (cmp. Table 3) with other IMU-based datasets, we have a high number of tracked body points. Furthermore, our number of participants, tasks, and samples are in the middle between the high and low number datasets. The category where we score the lowest compared to the other datasets is the total recording time, as we only record 1.5 hours in total. The comparison shows the trade-off we chose for these datasets, instead of maximizing a single category of the dataset, we decided to spread our resources across all categories to get a good variety in all of them.

**Table 3:** Overview of related gait datasets using IMU sensors for motion tracking, sorted by publication year.

Source	IMU Tracked Points	Participants	Tasks	Samples	Recorded Time	Publication Year
Khandelwal *et al*.^46^	4	20	7	51	6h	2017
Chereshneve *et al*.^47^	6	18	12	2111962	10h	2018
Truong *et al*.^48^	2	230	1	40.000	8.5h	2019
Loose *et al*.^49^	3	108	6	1080	30h	2019
Luo *et al*.^50^	6	30	9	1710	8h	2020
Losing *et al*.^51^	17	20	3	180 - 300	9h	2022
**CeTI-Locomotion**^34^	**17**	**50**	**5**	**4672**	**1.5h**	**2024**

## Usage Notes

The motion data and additional metadata are stored as tab-separated values (.tsv) and JSON (.json) files, and are as such compatible with a wide range of software applications and programming languages, thus promoting interoperability and seamless integration in different environments, as well as data processing efficiency. We recommend to use the processed data in *derivates/cut_segments* for analysis and classification, as the single repetitions already contain a lot of information. For the comparison of the classification of the identity or the actions performed, the code provided for the verification experiments can be used as a simple baseline. For the BIDS standard^36^ there are specialized libraries, such as PyBIDS^44,45^, for importing and processing the data.

## Data Availability

The custom code used for processing and technical validation are available along with the associated dataset^34^. The required libraries to execute the custom scripts have been included in the files *requirements.txt* and *requirements.yaml*. These files allow for the installation of the necessary libraries either directly via The Python Package Index (PyPI, https://pypi.org) or via the Anaconda software distribution (2020.11, https://www.anaconda.com). The script *preprocess_data.py* encompasses all the data processing steps that were performed subsequent to the export from Rokoko studio. To facilitate the replication of the technical validation, the script *verification_experiments.py* has been provided. This script allows for the execution of the technical validation on the processed data. Furthermore, the script *render_sequence.py* allows for the rendering the position data from *_motion.tsv files for visual analysis and verification. For additional details on the usage and execution of the custom code, please refer to the *README* file, which provides comprehensive instructions and guidelines.
